# Artificial intelligence–assisted quantification of fundus tessellation in early-onset high myopia

**DOI:** 10.3389/fmed.2025.1663903

**Published:** 2026-01-08

**Authors:** Yi-Ming Guo, Kexin Wu, Juan Huang, Bo Ma, Lu Ye

**Affiliations:** 1 Shaanxi Eye Hospital, Xi’an People’s Hospital (Xi’an Fourth Hospital), Affiliated People’s Hospital of Northwest University, Xi'an, China; 2 Department of Ophthalmology, The First Affiliated Hospital of Xi’an Jiaotong University, Xi'an, China

**Keywords:** artificial intelligence, axial elongation, early-onset high myopia, fundus tessellation density, pediatric myopia

## Abstract

**Purpose:**

To quantitatively evaluate fundus tessellation density (FTD) in children with early-onset high myopia (eo-HM) using artificial intelligence (AI)–assisted image analysis and to explore its association with axial length (AL).

**Methods:**

This cross-sectional study included children aged ≤6 years with eo-HM, defined as spherical equivalent (SE) ≤ −6.00 D and/or AL > 26.00 mm. Cycloplegic refraction, AL measurement, and ultra-widefield fundus photography were performed. A deep learning–based algorithm quantified FTD across concentric macular zones (1 mm, 3 mm, 6 mm) and anatomical sectors. Correlation and multivariate regression analyses were used to assess associations between FTD and AL.

**Results:**

A total of 47 eyes from 31 children were analyzed. Mean SE was −9.35 ± 3.74 D and AL was 25.70 ± 1.50 mm. FTD declined from center to periphery (*p* < 0.001), with greater values in nasal and inferior sectors at 6 mm. Overall FTD correlated with AL (*r* = 0.46, *p* = 1.47 × 10^−3^). In univariate regression, FTD significantly predicted AL (*β* = 18.16, *p* = 1.76 × 10^−5^, *R*^2^ = 0.352). Multivariable analysis showed that FTD in the 6 mm nasal and superonasal sectors remained independently associated with AL after adjusting for age and sex.

**Conclusion:**

AI-assisted quantification of FTD provides a sensitive and objective measure of early retinal changes in eo-HM. Regional FTD, especially in nasal and perifoveal regions, is strongly associated with axial elongation and may serve as a useful biomarker for early diagnosis and monitoring of pediatric high myopia.

## Introduction

Early-onset high myopia (eo-HM) is a distinct and clinically significant subtype of myopia, defined by a spherical equivalent (SE) of ≤ − 6.00 diopters or axial length (AL) > 26 mm, with onset occurring at or before six years of age (1). Although the prevalence of eo-HM among preschool children remains relatively low—estimated at 0.1% in China, 0.2% in Japan, and 0.6% in Singapore (2)—this condition warrants particular attention due to its early onset, rapid progression, and elevated risk of serious ocular complications, including retinal detachment, macular holes, and choroidal neovascularization, all of which may ultimately lead to irreversible vision loss (3, 4).

Atypical or subtle fundus changes are frequently observed in eo-HM, complicating early diagnosis. These include retinal thinning, scattered subtle lesions, or an indistinct tessellated fundus pattern; some cases may even show no obvious abnormalities. For example, in a cohort of 54 individuals with ARR3 gene mutations, Wang et al. reported that six female patients exhibited a morphologically normal posterior pole without signs of myopic retinal degeneration (classified as C0), while 64.8% (35/54) presented with characteristic tessellation and visible choroidal vessels (classified as C1) (5). The heterogeneous and frequently subtle nature of such changes highlights the need for more sensitive, objective, and reproducible tools for early detection and risk assessment in this vulnerable population.

Fundus tessellation (FT), characterized by increased visibility of large choroidal vessels due to thinned retinal pigment epithelium and choriocapillaris, represents an early and quantifiable imaging feature of myopic structural changes (6). Longitudinal cohort studies have demonstrated that FT may precedes more advanced forms of myopic maculopathy, such as lacquer cracks and diffuse chorioretinal atrophy (7). Supporting evidence comes from the 10-year follow-up of the Beijing Eye Study, which reported that 19% of highly myopic eyes with baseline FT progressed to myopic maculopathy (8). Accordingly, FT has been incorporated into international classification systems as a hallmark of early-stage myopic maculopathy and may serve as a useful imaging biomarker for disease severity and prognosis (9).

The advent of artificial intelligence (AI)–based image analysis has significantly advanced the automated and quantitative evaluation of fundus features. Recent studies have shown the value of AI in quantifying fundus tessellation density (FTD) and exploring its relevance to myopia. For instance, Li et al. analyzed FTD in 1,084 university students using a deep learning algorithm and found significant associations between FTD and AL, subfoveal choroidal thickness (SFCT), and vessel density in both the choriocapillaris and deeper choroidal layers (10). Similarly, Huang et al. assessed FTD in over 1,000 children and demonstrated its high prevalence across multiple retinal regions, while He et al. observed a stage-wise decline in FTD with increasing pathological myopia severity (11, 12). These findings collectively support the use of AI-assisted fundus imaging as a promising approach for early detection, classification, and longitudinal monitoring of myopia-related structural changes.

Therefore, this study aims to utilize AI-assisted fundus image analysis to quantitatively assess FTD in children with eo-HM. By characterizing the spatial distribution and magnitude of FTD, we seek to elucidate early retinal structural alterations in this population and evaluate the potential of FTD as a non-invasive biomarker for early identification, risk stratification, and disease monitoring in pediatric high myopia.

## Method

### Study population

This cross-sectional study was conducted at the Department of Ophthalmology and the Optometry Center of Xi’an People’s Hospital (Xi’an Fourth Hospital) between January 2023 and December 2024. The study protocol was approved by the Ethics Committee of Xi’an Fourth Hospital (Approval No. 20230011) and adhered to the tenets of the Declaration of Helsinki. Written informed consent was obtained from the parents or legal guardians of all participating children, and verbal assent was obtained from the children prior to examination.

Children were eligible for inclusion if they met the diagnostic criteria for eo-HM, defined as: (1) presentation during the preschool period (typically ≤6 years of age; note that although onset occurred before age 6, some children underwent complete ophthalmic examinations at later visits due to limited cooperation in early childhood or delayed clinical referral); (2) SE ≤ −6.00 diopters and/or AL > 26.00 mm; (3) absence of known ocular or systemic pathology; and (4) no history of hereditary ocular diseases (e.g., congenital cataract, retinal dystrophy), ocular trauma, or prior refractive surgery.

### Ophthalmic examinations

All participants underwent standardized ophthalmologic examinations. To ensure accurate assessment of refractive status, cycloplegic refraction was performed using 1.1% atropine sulfate ophthalmic gel, administered three times daily for three consecutive days. Refraction measurements were obtained on the fourth day using an automated refractometer (ARK-1, NIDEK, Japan). SE was calculated as the spherical power plus half of the cylindrical power.

AL was measured using the IOLMaster 500 (Carl Zeiss Meditec AG, Germany). Ultra-widefield fundus imaging was performed using the Optos retinal imaging system (Optos, United Kingdom). All procedures were conducted by trained ophthalmologists and optometrists in accordance with standardized protocols to ensure consistency and data reliability.

### Extraction of FTD using AI-based technology

FTD was quantified using a deep learning–based fundus image analysis system (EVision AI, Eaglet Vision Technology Co., Ltd.). All fundus photographs underwent dual-level quality control, including (1) clinical image evaluation performed by trained ophthalmic staff following standard retinal photography protocols (assessing clarity, fixation stability, illumination uniformity, and absence of artifacts), and (2) automated system-level assessment based on the manufacturer’s built-in criteria for image clarity, illumination balance, and signal-to-noise ratio (SNR). Images failing to meet these vendor-recommended thresholds were automatically excluded before FTD computation.

The image processing pipeline comprised four primary stages: preprocessing, anatomical localization, semantic segmentation, and quantitative analysis. During preprocessing, regions of interest (ROIs) centered on the optic disc and macula were automatically identified and cropped. Image brightness and color balance were normalized to reduce inter-sample variability. Contrast-limited adaptive histogram equalization (CLAHE) was applied to enhance local contrast and improve visualization of choroidal vasculature.

Anatomical landmarks were identified using a hybrid model that integrated ResNet50 with edge detection algorithms for optic disc and macular localization. Choroidal vessel segmentation was conducted using a TransUnet-based architecture, which combines transformer modules with convolutional encoders to enable high-resolution, context-aware semantic segmentation. This pipeline has been previously validated in multiple peer-reviewed studies employing the same commercial analysis system, demonstrating stable segmentation performance in large-scale retinal datasets (13, 14).

**Figure 1:**
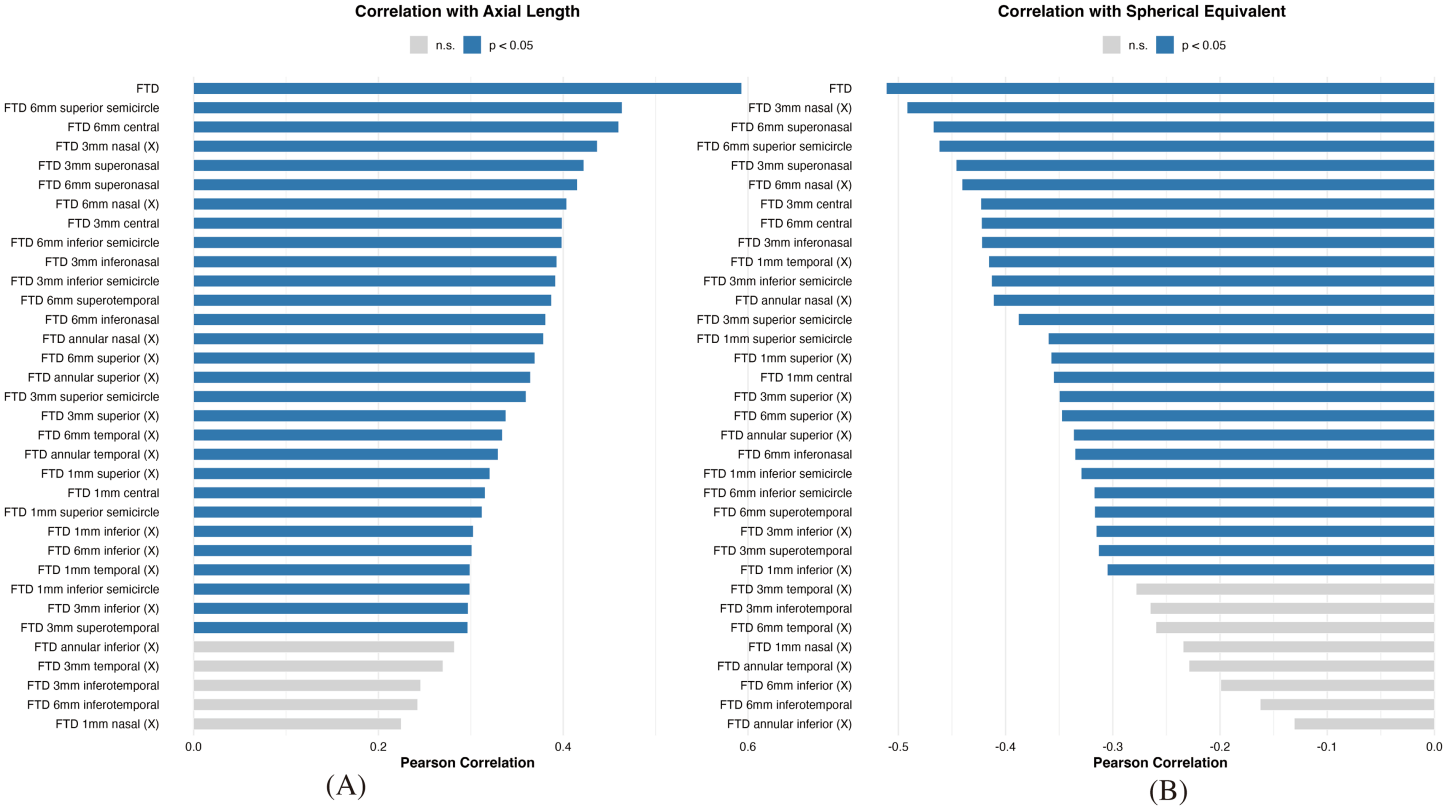
Pearson correlation between regional FTD and ocular biometric parameters in preschool children with early-onset high myopia. Bar plots summarize the Pearson correlation coefficients between FTD values in 34 macular sectors and **(A)** axial length or **(B)** spherical equivalent. Each bar represents a specific sector of the macula, arranged from weakest to strongest correlation. Bars are color-coded according to statistical significance (*p* < 0.05). Blue bars indicate statistically significant associations; gray bars denote non-significant results. Stronger correlations were observed between regional FTD and axial length than with SE, particularly in mid-peripheral sectors.

FTD was defined as the average area of visible choroidal vessels per unit fundus area within the ROI. In addition to the overall FTD, computed within concentric circles centered on the macula (diameters of 1 mm, 3 mm, and 6 mm), regional FTD was quantified using both orthogonal (“+”) and oblique (“×”) sector-based division methods. These included vertical (superior/inferior), horizontal (nasal/temporal), and oblique quadrants (superonasal, inferonasal, superotemporal, inferotemporal), allowing for detailed spatial characterization of tessellation patterns (Figure 1).

### Statistical analysis

All statistical analyses were performed using R software (version 4.3.2; R Foundation for Statistical Computing, Vienna, Austria). Continuous variables were tested for normality using the Shapiro–Wilk test and reported as mean ± standard deviation (SD). Categorical variables were summarized as counts and percentages. Cases with incomplete biometric data or substandard image quality (e.g., motion blur, low contrast) were excluded from analysis.

Differences in FTD across macular zones and sectors were evaluated using paired-sample *t*-tests or one-way repeated measures ANOVA, as appropriate. Spatial asymmetry of FTD was assessed by comparing superior vs. inferior and nasal vs. temporal regions.

Pearson correlation coefficients were calculated to evaluate associations between FTD and ocular biometric parameters (AL and SE). Univariate and multivariate linear regression analyses were conducted to further explore these relationships, with AL and SE as dependent variables and regional FTD values as independent predictors. Covariate multicollinearity was assessed using variance inflation factors (VIFs), with a threshold of <5 considered acceptable. All regression models showed low multicollinearity, with VIF values ranging from 1.00 to 1.19. To account for the potential non-independence between fellow eyes, we additionally examined inter-eye correlation using generalized estimating equations (GEE). The results were consistent with the primary regression models, suggesting that intra-subject clustering did not materially affect the observed associations.

A two-tailed *p*-value < 0.05 was considered statistically significant. All outcomes were reported with appropriate summary statistics, including regression coefficients (*β*), 95% confidence intervals (CI), *R*^2^ values, and corresponding *p*-values.

## Result

### Demographic and clinical characteristics

A total of 31 children with eo-HM were included in the final analysis. After excluding 15 eyes due to insufficient image quality or incomplete clinical data, 47 eyes were retained for quantitative assessment. Among the participants, 19 (61.29%) were male and 12 (38.71%) were female, with a mean age of 7.28 ± 2.31 years (range: 5.9–9.1 years). Of the included eyes, 22 (46.81%) were right eyes and 25(53.19%) were left eyes. The mean AL was 25.70 ± 1.50 mm, and the mean SE was −9.35 ± 3.74 D. Detailed clinical and demographic data are presented in Table 1.

**Table 1:** Demographic and clinical characteristics.

Characteristic	[ALL]	Number of subjects
	*N = 47*	
Gender		31
Female	12 (38.71%)	
Male	19 (61.29%)	
Age (years)	7.28 ± 2.31	31
Eye side		47
OD	22 (46.81%)	
OS	25 (53.19%)	
Axial length (AL, mm)	25.70 ± 1.50	47
Spherical equivalent (SE, D)	−9.35 ± 3.74	47

### Distribution and regional patterns of FTD

FTD was successfully quantified in all 47 eyes using an automated algorithm previously validated for fundus image analysis. The mean overall FTD was 0.08 ± 0.05. Across macula-centered concentric zones, FTD demonstrated a clear decreasing gradient from center to periphery: 0.28 ± 0.22 in the 1 mm zone, 0.21 ± 0.19 in the 1.5 mm zone, 0.16 ± 0.16 in the 3 mm zone, and 0.13 ± 0.11 in the 6 mm zone (*p* < 0.001), indicating centripetal attenuation of tessellation. The presence of patchy atrophic lesions did not significantly influence the mean FTD, suggesting minimal impact on the robustness of FTD quantification.

To investigate spatial heterogeneity, both orthogonal (“+”) and oblique (“×”) segmentation methods were applied to the 1 mm, 3 mm, and 6 mm regions. No significant asymmetry was observed in the central 1 mm or 3 mm zones. However, in the 6 mm zone, the orthogonal analysis revealed significantly higher FTD in the inferior hemisphere compared to the superior (0.16 ± 0.12 vs. 0.11 ± 0.12, *p* < 0.001). Among quadrants, the inferonasal region exhibited the highest FTD (0.20 ± 0.17), while the superotemporal quadrant had the lowest (0.10 ± 0.11, *p* = 0.004). The oblique method similarly showed higher FTD in the nasal sectors than temporal sectors (0.19 ± 0.20 vs. 0.10 ± 0.12, *p* = 0.015), indicating preferential tessellation accumulation in the nasal and inferior perifoveal retina (Table 2).

**Table 2:** Regional distribution of FTD across macular zones and sectors.

Zone (macula-centered)	Region	O-method mean ± SD	“+”-method *p*-value	Region	X-method mean ± SD	“×”-method *p*-value
1 mm zone						
			0.86	Superior quadrant	0.43 ± 0.25	0.78
	Superior hemisphere	0.3 ± 0.24		Inferior quadrant	0.34 ± 0.24	
	Inferior hemisphere	0.29 ± 0.22		Nasal quadrant	0.27 ± 0.23	
				Temporal quadrant	0.31 ± 0.24	
3 mm zone						
						
	Superior hemisphere	0.18 ± 0.17	0.78			
	Inferior hemisphere	0.17 ± 0.17				
	Superonasal quadrant	0.2 ± 0.19	0.42	Superior quadrant	0.21 ± 0.21	0.507382388
	Inferonasal quadrant	0.22 ± 0.19		Inferior quadrant	0.23 ± 0.2	
	Superotemporal quadrant	0.19 ± 0.19		Nasal quadrant	0.19 ± 0.19	
	Inferotemporal quadrant	0.19 ± 0.19		Temporal quadrant	0.15 ± 0.16	
6 mm zone						
	Superior hemisphere	0.11 ± 0.12	<0.001			
	Inferior hemisphere	0.16 ± 0.12				
	Superonasal quadrant	0.14 ± 0.16	0.0045	Superior quadrant	0.11 ± 0.13	0.015
	Inferonasal quadrant	0.2 ± 0.17		Inferior quadrant	0.17 ± 0.14	
	Superotemporal quadrant	0.1 ± 0.11		Nasal quadrant	0.19 ± 0.2	
	Inferotemporal quadrant	0.13 ± 0.15		Temporal quadrant	0.1 ± 0.12	

### Correlation and regression analyses

**Figure 2:**
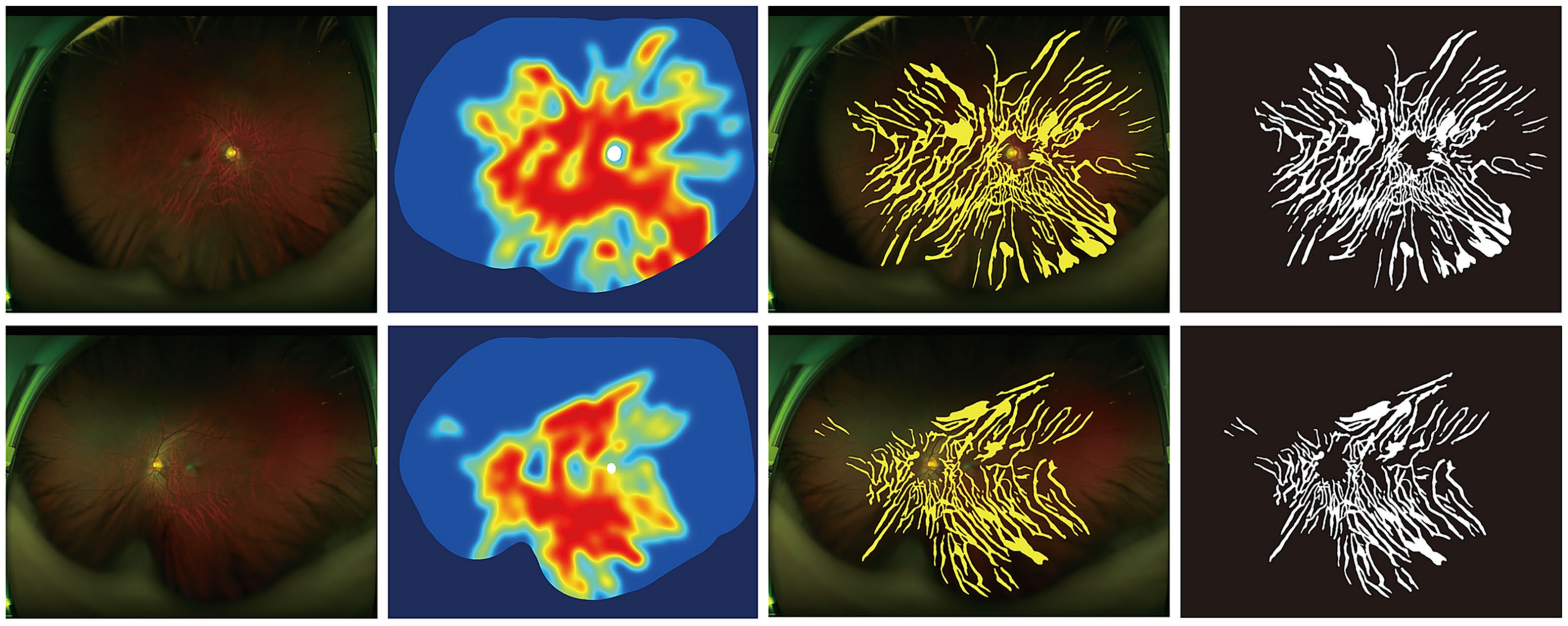
Quantitative visualization of fundus tessellation density in early-onset high myopia. Each column represents a distinct imaging modality: (1) Original fundus photograph; (2) FTD heatmap indicating the intensity of exposed choroid; (3) tessellation overlay demonstrating the spatial distribution of tessellated patterns; (4) binary segmentation map used for quantitative analysis. The top and bottom rows correspond to the right and left eyes of a representative EOHM case, respectively.

Pearson correlation analyses demonstrated significant associations between FTD and ocular biometric parameters, particularly within the 6 mm perifoveal region. Overall FTD was positively correlated with AL (*r* = 0.46, *p* = 1.47 × 10^−3^) and negatively correlated with SE (*r* = −0.42, *p* = 3.10 × 10^−3^), indicating that greater tessellation was associated with axial elongation and higher myopic refractive error (Figure 2).

**Figure 3:**
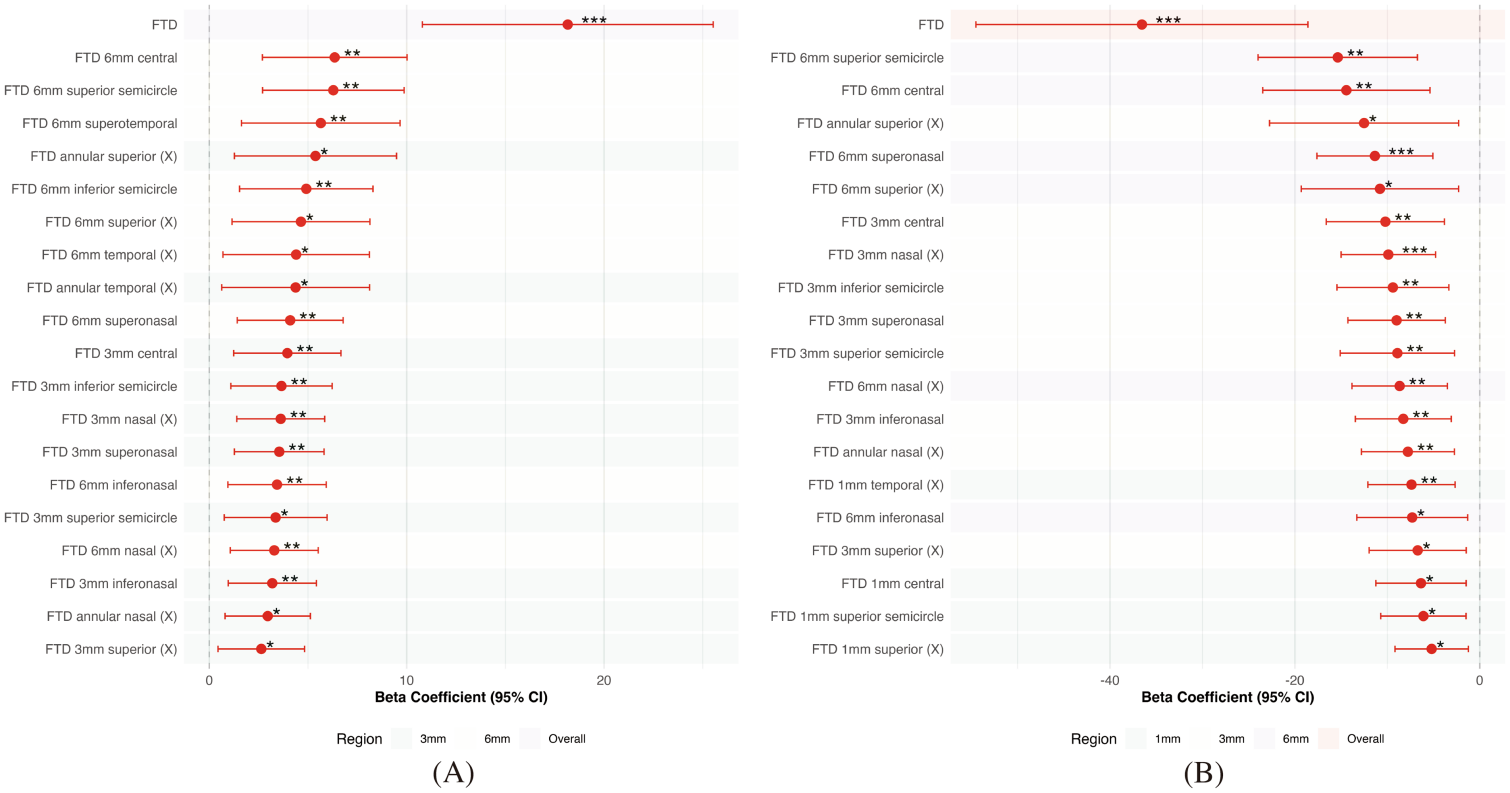
Regional associations between FTD and ocular biometric parameters across macular sectors. **(A)** Univariate linear regression coefficients (*β* values) for the association between FTD in different macular regions and AL. **(B)** Univariate regression coefficients for the association between regional FTD and SE. The 6 mm perifoveal zone, especially the superonasal, nasal, and inferior sectors, exhibited the strongest associations with both AL elongation and myopic refractive error. Bars represent β coefficients, and error bars denote 95% confidence intervals. Asterisks indicate statistically significant associations (*p < 0.05, **p < 0.01, ***p < 0.001).

Univariate linear regression further supported these findings. Overall FTD was a significant predictor of AL (*β* = 18.16, *p* = 1.76 × 10^−5^, *R*^2^ = 0.352) and SE (*β* = −36.55, *p* = 2.4 × 10^−4^, *R*^2^ = 0.261). Subregional analyses identified the 6 mm zone as having the highest predictive value. Specifically, FTD in the superonasal quadrant of the 6 mm region was significantly associated with both AL (*β* = 4.10, *p* = 4.6 × 10^−3^, *R*^2^ = 0.172) and SE (*β* = −11.34, *p* = 9.3 × 10^−4^, *R*^2^ = 0.218). Additional associations were observed in the nasal and superior subfields, further highlighting the spatially heterogeneous relationship between FTD and ocular biometry (Figure 3).

Multivariable regression analyses, adjusted for age and sex, confirmed that FTD in the 6 mm perifoveal region—particularly in the nasal and temporal sectors—remained independently associated with AL and SE. These findings suggest that peripheral FTD may serve as a sensitive surrogate marker for early axial elongation. In contrast, no significant associations were found within the central 1 mm zone, possibly reflecting the relative structural preservation of the foveal region in early stages of eo-HM (Table 3).

**Table 3:** Correlation and regression analyses between FTD and ocular biometric parameters.

Variable	estimate_3mm	p_3mm	estimate_6mm	p_6mm
FTD central	4.26	0.001	6.13	0.001
FTD superior semicircle	3.27	0.009	5.44	0.001
FTD inferior semicircle	4.30	0.001	4.13	0.011
FTD superonasal	4.11	0.000	2.27	0.106
FTD inferonasal	3.06	0.005	4.69	0.000
FTD superotemporal	2.95	0.017	3.56	0.001
FTD inferotemporal	2.12	0.065	3.23	0.007
FTD superior (X)	3.10	0.005	1.83	0.212
FTD inferior (X)	2.09	0.052	7.01	0.000
FTD nasal (X)	3.89	0.000	5.63	0.004
FTD temporal (X)	2.88	0.031	3.74	0.031

Collectively, these results underscore the value of spatial FTD analysis in capturing subtle, region-specific structural alterations associated with eo-HM. Quantitative assessment of FTD may provide novel imaging biomarkers for early disease detection and individualized risk stratification.

## Discussion

The progression of high myopia is often accompanied by gradual structural alterations of the posterior segment, including choroidal thinning, retinal atrophy, and macular degeneration. These degenerative changes are among the leading causes of irreversible visual impairment. Fundus tessellation, a common and early ocular manifestation in myopic individuals, shows marked variation in prevalence across geographical regions, age demographics, and refractive conditions. In 2014, Yoshihara et al. reported FT changes in 43% of 100 healthy adults aged 22–39 years in Japan, with 16 individuals classified as having strongly tessellated fundus (15). In China, Guo et al. reported a 48.1% prevalence of FT among 1,443 children aged 9–16 years in Beijing (16). More notably, a 2021 study involving 513 children and adolescents with high myopia found that only 5.7% showed no FT changes, suggesting that FT is highly prevalent in the pediatric high myopia population and closely associated with ocular biometric parameters (17). Previous studies have further indicated a strong relationship between FT and axial elongation. Yamashita et al. found that individuals with FT had significantly longer axial lengths compared to non-tessellated counterparts (15). Subsequent analyses revealed that the location of tessellation also matters: FT concentrated in the posterior pole was associated with longer axial lengths compared to peripapillary or inferiorly distributed FT (18). Gong’s study in children with low myopia further delineated this relationship, demonstrating that as FT severity increased, choroidal thickness decreased, and higher tessellation grades were independently associated with greater corneal radius of curvature (19). FT has also been proposed as an early manifestation in the spectrum of myopic maculopathy, with some individuals demonstrating progression during follow-up to advanced complications such as lacquer crack formation and diffuse chorioretinal atrophy (7). In a 10-year longitudinal study of 2,695 high myopia cases in Beijing, 19% of eyes exhibiting tessellated fundi at baseline progressed to definite myopic maculopathy at follow-up (8). These findings collectively support the prognostic value of FT in myopic retinal risk assessment.

Early-onset high myopia, characterized by rapid axial elongation and early disease onset, presents a unique challenge. Retinal changes at this stage are often subtle and nonspecific, limiting the sensitivity of traditional fundus evaluation. In this context, image-based quantitative analysis offers an emerging approach for detecting early morphological changes. In 2021, Shao et al. introduced a deep-learning-based pipeline for quantifying FTD from color fundus images. This automated workflow, initially applied to community-dwelling adults, demonstrated that FTD was significantly associated with axial length, age, sex, choroidal thickness, and peripapillary atrophy (13). Subsequent studies expanded the method to university students (10) and school-aged children (20), further validating the reproducibility and applicability of FTD quantification across populations. Notably, FT patterns in the macular region were shown to correlate closely with axial elongation and spherical equivalent, suggesting that spatial characteristics of FT may reflect distinct pathological trajectories of myopia progression (14). Furthermore, the association between FTD and choroidal vascular density suggests a potential role for FTD as a biomarker of choroidal perfusion and early-stage degeneration (10).

Building upon these foundations, the present study is the first to apply AI-driven quantitative FTD analysis to a cohort of children with eo-HM. By integrating clinical profiles with image-derived metrics, we characterized the distribution and severity of FT in this population. Overall, we observed relatively low FTD values across the cohort, indicating that widespread fundus degeneration may not yet be prominent in most eo-HM cases, possibly due to the relatively young age of participants and the early stage of ocular development, during which structural alterations remain subclinical or subtle (21, 22). Interestingly, in eyes exhibiting patchy or diffuse atrophic changes, overall FTD values remained within a relatively stable range. This may reflect compensatory integrity in unaffected retinal regions or signal interference from residual retinal pigment epithelium (RPE) pigmentation (23, 24). Compared with studies in older children and adults, the lower overall FTD observed in our eo-HM cohort is likely influenced by age-related factors such as denser melanin pigmentation and relatively preserved RPE–choroid integrity, which reduce choroidal visibility on color fundus imaging and consequently yield smaller quantitative FTD values (10, 14). Additionally, inter-individual differences in pigmentation density and anatomical features could influence the quantification outcomes (25). Despite the overall low and stable FTD levels, we identified a consistent spatial gradient: FTD values were highest in the central macular region and progressively decreased with radial distance. This suggests that the macula may represent an early site of pathological change in eo-HM, with FTD offering a sensitive metric for detecting incipient abnormalities (26). Therefore, FTD should not be interpreted solely as an index of atrophic severity, but rather as a composite biomarker reflecting regional anatomical context, developmental stage, and potential pathological remodeling processes (14).

To further elucidate the spatial characteristics of FTD in eo-HM, we employed both cross (“+”) and oblique (“×”) quadrant-based segmentation analyses of the macular region (1 mm, 3 mm, and 6 mm zones). Although no significant differences were observed in the 1 mm and 3 mm zones, analysis of the 6 mm region revealed significantly higher FTD in the inferior hemisphere compared to the superior hemisphere. Additional four-quadrant analyses demonstrated the highest FTD in the inferonasal quadrant and the lowest in the superotemporal quadrant. The “×”-shaped segmentation confirmed a consistently higher FTD in the nasal region. These regional differences are consistent with previous reports and may reflect the complex interplay of biomechanical stress, local vascular supply, and anatomical variation (10). Anatomically, the optic nerve head is located nasally, and the peripapillary region may be more susceptible to early mechanical stretching during axial elongation (23). Furthermore, physiological differences in choroidal perfusion—particularly reduced blood flow in the inferior retina—may predispose these areas to early degenerative changes (27). Additional factors, such as variability in RPE metabolic demand, local stress response, and asymmetrical developmental rates across retinal regions, may also contribute to the observed directional bias of FT accumulation (28). Importantly, the choice of segmentation strategy may influence the interpretation of spatial FT patterns. The “+”-shaped model is commonly used in the evaluation of RNFL defects in glaucoma, while the “×”-shaped model is suited for analyzing peripapillary morphology (29). Our findings underscore the importance of considering region-specific FTD assessments when evaluating early pathological changes in eo-HM, as different retinal zones may experience heterogeneous mechanical and metabolic burdens.

Beyond topographic trends, our correlation and multivariable regression analyses consistently demonstrated that increased FTD, particularly in the 6 mm perifoveal region, was significantly associated with axial elongation and greater myopic refractive error. These associations remained robust after adjusting for age and sex in multivariable models, highlighting the independent contribution of regional FTD to ocular structural changes. Notably, the superior and central sectors within the 6 mm zone exhibited the strongest associations with axial length, with effect sizes surpassing those in corresponding 3 mm regions, suggesting a spatial gradient wherein more peripheral FTD alterations may reflect heightened susceptibility to axial elongation. Besides, the absence of correlation in the central macular zone should be interpreted cautiously, as early foveal structure is often preserved in young children and central FTD typically exhibits lower variability compared with perifoveal regions. Age emerged as a stable and significant covariate across all models, whereas sex showed no significant influence, indicating that the observed associations were not confounded by sex differences. Collectively, these findings underscore the regional specificity and clinical relevance of perifoveal FTD as a potential topographic imaging biomarker for early axial remodeling in pediatric high myopia, offering novel insights into spatially patterned retinal changes associated with early disease progression.

Despite these contributions, our study has several limitations. First, the sample size was relatively small, particularly for eo-HM, which inevitably limits the statistical power of regional FTD analyses and increases the risk that subtle associations may remain undetected. Larger, multicenter cohorts will be essential to enhance the stability, robustness, and population generalizability of these findings. In addition, external validation in independent pediatric cohorts will be crucial to further confirm the diagnostic and monitoring value of AI-derived FTD metrics. Second, the image quality of fundus photographs was occasionally suboptimal owing to limited patient cooperation, particularly among younger children, leading to missing data in optic disc-centered images and potentially reducing the accuracy of FTD calculations. Future studies should aim to expand the cohort and improve image quality to enhance data robustness. Moreover, as a cross-sectional study, our findings are inherently limited to associative interpretations and cannot establish causality between FT features and disease progression. Longitudinal follow-up studies will be necessary to validate FTD as an early biomarker of pathological changes and to determine its predictive value in the natural history of eo-HM. Finally, our current analysis focused exclusively on color fundus photographs. Integration of multimodal imaging (e.g., OCT, OCTA) and comprehensive clinical data (e.g., questionnaire-based risk factors) will be essential for a more comprehensive understanding of the relationship between FT and eo-HM pathophysiology.

In conclusion, our study presents the first application of automated fundus tessellation quantification specifically in children with early-onset high myopia eo-HM, a rare and clinically distinct subgroup characterized by rapid axial elongation and a high genetic burden. In addition, by mapping the spatial patterning of FTD at a developmental stage when conventional fundus changes are often absent, this study provides the earliest objective imaging evidence of region-specific retinal vulnerability in the natural history of high myopia—an aspect that has not been demonstrated in any previous FTD-related research. The results reveal both stable global FTD patterns and region-specific variations that may signal early pathological remodeling. These findings support the potential of FTD as a non-invasive, quantifiable imaging marker for early-stage retinal changes in high myopia. Clinically, the potential value of FTD as an early imaging biomarker lies in its ability to provide an objective, region-specific index of posterior pole integrity before overt pathological changes emerge. In practical application, longitudinal monitoring of perifoveal FTD—particularly in the 6 mm nasal and inferior regions—may help identify children at higher risk of accelerated axial elongation and thus inform closer follow-up intervals. Although unified operational cut-offs for screening are not yet available, existing pediatric studies consistently indicate that progressive increases in regional FTD, rather than absolute values alone, may serve as a clinically meaningful trigger for intensified surveillance. Overall, this study thereby highlights the clinical utility of quantitative FTD profiling in pediatric screening, individualized risk stratification, and longitudinal monitoring strategies.

## Data Availability

The raw data supporting the conclusions of this article will be made available by the authors, without undue reservation.
